# The impact of the Choice on Termination of Pregnancy Act of 1996 (Act 92 of 1996) on criminal abortions in the Mthatha area of South Africa

**DOI:** 10.4102/phcfm.v1i1.36

**Published:** 2009-07-14

**Authors:** Banwari L. Meel, Ram P. Kaswa

**Affiliations:** 1 Faculty of Health Sciences, Walter Sisulu University, South Africa

**Keywords:** South Africa, foetuses, criminal abortion, termination, Choice on Termination of Pregnancy Act of 1996 (Act 92 of 1996)

## Abstract

**Background:**

The Choice on Termination of Pregnancy Act of 1996 (Act 92 of 1996) allows abortions to be legally carried out in South Africa. It is not clear how many people are utilising this service. Mthatha is a poverty-stricken area with a high rate of illiteracy. The available infrastructure, such as roads, health facilities and communication, is poor.

**Method:**

This was a retrospective, descriptive study carried out at the Nelson Mandela Academic Hospital in Mthatha. The registered criminal abortion cases recorded between 1993 and 2006 were analysed.

**Results:**

There were 51 cases of criminal abortions recorded from 1993 to 2006. Of these, 32 were aborted in the first trimester of pregnancy and the rest were in the second trimester. No significant gender differences were observed among aborted babies. 10 of the foetuses were male and nine were female. The highest number (nine) of abortions was recorded in 1993 and in 2005. The highest number of criminal abortions (11) took place in May. Most cases (35) were concealed births and were discovered accidentally either by the public or the police.

**Conclusion:**

The Choice on Termination of Pregnancy Act of 1996 (Act 92 of 1996) had no impact on criminal abortions in the Mthatha area of South Africa.

## INTRODUCTION

A total of 26 million legal and 20 million illegal abortions were performed throughout the world in 1995.^1^ Between 1996 and 1999, public sector clinics in South Africa performed 69 894 abortions.^2^ Worldwide, more than 500 000 women and girls die of complications related to pregnancy and childbirth every year. More than 99% of these deaths occur in developing countries such as South Africa.^3^ Incomplete abortions and, in particular, unsafe abortions are important causes of mortality and morbidity in South Africa.^4^ In view of this high number of deaths related to pregnancy, the South African Choice on Termination of Pregnancy Act (Act 92 of 1996 – herein referred to as the Act) was promulgated in 1996, and this has given women the right to terminate a pregnancy on request. The Act has been associated with a massive reduction in women presenting with incomplete abortions.^5^

The Act liberalised abortions in South Africa. Whilst an appreciable number of terminations of pregnancies (TOPs) have already been performed in terms of the Act, it has also surfaced that an array of factors of various kinds may impede its further implementation and operation.^6^ The Act aims to promote female reproductive autonomy through legitimising free access to abortion up to 20 weeks of gestation. An unacceptably high rate of unsafe abortion prevails, particularly in rural areas and amongst adolescents.^7^ The legalising of TOPs alone cannot ensure the implementation of abortion services. There are still more ‘technically illegal’ abortions than legal ones in South Africa.^8^ In South Africa, extensive media coverage prior to the passage of the law ensured almost universal awareness of the Act, but little public education took place at the same time. Under the Act, abortion services can now theoretically be implemented, even in conservative rural areas, but successful, practical implementation of these services is still a work in progress..^9^ Some professional nurses do not want to work in TOP services and do not want to be associated with those who render these services.^10^

There is an enormous amount of literature in the lay press on abortion, but only a few published studies****. There are no published articles about criminal abortions in the Mthatha area of South Africa. People in the area are generally poor and live on very scarce resources. Mthatha (Umtata) was the capital of the former black homeland of Transkei, which is now incorporated into the Eastern Cape Province. The Eastern Cape has a population of seven million, of whom nearly four million inhabit the Transkei region. Almost three quarters (74%) of the province's population earns less than R1 500 per month, and 41% of households have an income of under R500 per month. The Eastern Cape has the country's second-highest proportion of the poor (44.5%), with the equivalent figure in the Transkei no less than 92%.^11^ The Nelson Mandela Academic Hospital mortuary is the only mortuary in the area, serving a population of about 300 000. The purpose of this study was to highlight the problem of abortions in the Mthatha area of South Africa.

## METHOD

This was a descriptive study. It reviewed all abortion cases registered in the mortuary of the Nelson Mandela Academic Hospital between January 1993 and December 2006 for medico-legal investigation. The police usually bring a mass of human tissue for determination of whether it is a foetus. The referrals are mainly from the Mthatha, Ngqeleni, Tsolo and Mquanduli magisterial districts. The month and year were recorded in all abortion cases. The genders of the foetuses were identified in some autopsies. All the records of the study period were reviewed, compiled and collated manually.

## RESULTS

**FIGURE 1:**
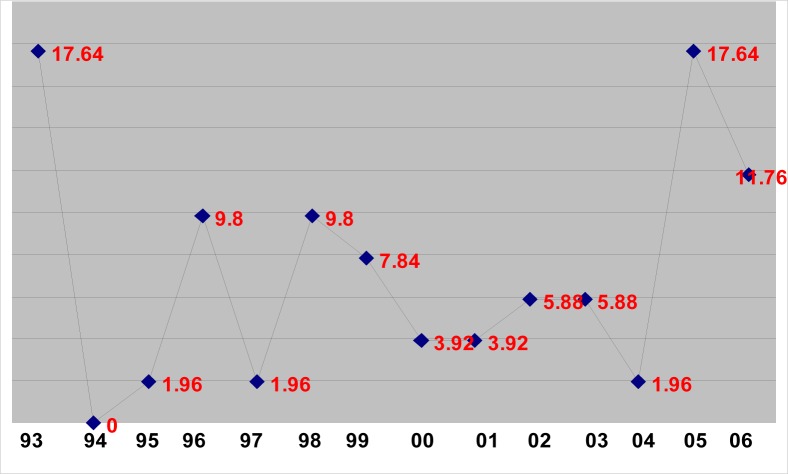
Yearly (1993-2006) distribution of criminal abortions in the Transkei region of South Africa (n=51)

There were 51 cases of criminal abortions recorded from 1993 to 2006 (Table 1 and Figure 1). The highest number of cases (nine; 17.64%) was recorded in 1993 and 2005. There was no record of any criminal abortions in 1994 (Table 1). There were 32 (62.74%) first-trimester abortions, and the rest (37.26%) were in the second trimester. There were no significant gender differences. 10 foetuses were male and nine were female. The rate of abortions were lowest (1) in April and December. The highest number of abortions (11; 21.56%) were carried out in May (Table 2). Most of the abortions (35; 68.62%) were concealed births and discovered accidentally either by the public or the police.

**TABLE 1:** Yearly distribution of criminal abortions in the Mthatha area of South Africa (1993-2006)

YEAR	ABORTIONS	PERCENTAGE
1993	9	17.64
		
1994	0	0
1995	1	1.96
1996	5	9.80
1997	1	1.96
1998	5	9.80
1999	4	7.84
2000	2	3.92
2001	2	3.92
2002	3	5.88
2003	3	5.88
2004	1	1.96
2005	9	17.64
2006	6	11.76

**AVERAGE**	**3.64**	**7.14**

**TABLE 2:** Monthly distribution of criminal abortions (1993-2006)

MONTH	ABORTIONS	PERCENTAGE
January	2	3.92
February	2	3.92
March	3	5.88
April	1	1.96
May	11	21.56
June	5	9.80
July	9	17.64
August	4	7.84
September	6	11.76
October	4	7.84
November	3	5.88
December	1	1.96

**AVERAGE**	**4.25**	**8.33**

## DISCUSSION

The Transkei region is one of the most rural parts of South Africa. The roads are in a state of disrepair and therefore access to these areas is difficult. The local chiefs solve most of the minor disputes. They pronounce the punishment according to the nature of the criminal act. Illegal abortions are not considered a serious crime, as they are limited to the person concerned and most of the time remain undetected. The amount of illegal abortions carried out is generally underestimated. 51 criminal abortions were brought to the notice of the medico-legal investigation team over the period 1993 to 2006. The public accidentally found most of the aborted material and informed the police. Some women who aborted had post-abortion complications such as haemorrhage and infection.

Traditional healers are consulted by over 70% of South Africans before they consult any other type of health care professional.^12^ Therefore, we could assume that at least some of those with complications from criminal abortions go to traditional healers. A lack of information on abortion rights under the Act and the perceived poor quality of the designated facilities are the most important barriers to access and therefore should be addressed by policymakers and health service management. The willingness of women to self-medicate and visit traditional healers in these circumstances may influence the overall ability of the new legislation to reduce abortion morbidity.^13^

The Abortion Act came into effect in January 1997. The highest number of cases (9, 17.64%) was recorded in 1993 (prior to the Act), and in 2005 (after the Act) (Table 1 and Figure 1). This shows that there was no decrease in criminal abortions before and after the promulgation of the Act. However, there is not enough evidence to justify the claim in this study. Backyard abortions, ending up in sepsis, are still carried out by unqualified persons.^4^ Criminal abortions have a significant correlation with fever, septic shock and septic abortion. Of all the pregnancies, 35.0% were unwanted and 27.1% of these were illegally terminated by induced abortions. Unwanted pregnancy is one of the most important risk factors for induced abortion.^14^ It is difficult to estimate the number of deaths in this study among the women who aborted, as they remained unrecognised and probably were treated by traditional healers. The lack of TOP clinics in rural areas has made many rural folk seek assistance from traditional healers, particularly as they are easily accessible. The traditional healers speak the patients’ language and give the patients a sense of comfort and wellbeing. The traditional healing system readily helps in dealing with the psychosocial stress resulting from abortion. Despite the legalisation of TOPs, these services remained stigmatised.^10^

Complications as a result of criminal abortions in the first trimester are much less than in advanced pregnancy. In this study, 32 (62.74%) foetuses brought for medico-legal attention were from early in the pregnancy, and 19 (37.26%) were from late in pregnancy. There was no observation of female feticide in this study. There were no gender-biased abortions in this area, as 10 foetuses (19.6%) were male and nine (17.4%) were females. The criminal abortion rate was at its lowest in the religious festival months of April and December. The religious months seem to be protective against criminal abortions, while they are highest in May (11; 21.56%) (Table 2). Although there is legislation in place to make criminal abortions unnecessary, its implementation is poor. This is because people do not fully accept it. The knowledge of this legal right among rural women is low. The confidentiality and secrecy of these TOPs are not well perceived by the community. A study carried out by Jewkes *et al*. showed that nearly two-thirds of women had self-induced abortions or had consulted a traditional healer. A minority of these women indicated that they did this because they experienced barriers to the use of legal services.^13^

The majority of women in Mthatha are not using TOP services. 54% of the women had not used legal services because they did not know about the law, while 15% knew of their legal rights but did not know of a legal facility.^13^ Others did know where to access legal services, but feared rude staff (17%) or breaches of confidentiality (6.5%). Some (6.5%) had been unable to get a legal abortion early enough in pregnancy to comply with the law.^13^ HIV/AIDS has complicated the issue of TOP in public facilities, as it attaches dual stigmatisation. As a result, women pay less attention to a woman who is in need of support not only for HIV, but also for an unwanted pregnancy.

### conclusion

The Act permits abortions to be done legally. The services are probably underutilised and underserved. This is a preliminary inquiry related to abortion in this area. It provides some insight that justifies expanded efforts to initiate and develop a programme for a detailed, comprehensive study.
